# STRATEGIES FOR PARTNERING WITH CARCERAL SETTING STAFF AND LEADERSHIP

**DOI:** 10.1093/geroni/igac059.1683

**Published:** 2022-12-20

**Authors:** Stephanie Grace Prost

**Affiliations:** University of Louisville, Louisville, Kentucky, United States

## Abstract

The mission of carceral agencies--the pursuit of public safety andsecurity--often conflicts with the primary aim of social justice-oriented scholarship, most notably research objectives related to health promotion in jails and prisons. However, such research is essential to awareness building, policy reformation, and revisions today-to-day practices that increase the health and well-being of historically marginalized populations. As a result, the conduct of aging research within carceral settings requires specialized knowledge and skills including frequent, targeted communication,transparency, humility, and flexibility. This presentation includes discussion of strategies for building and maintaining successful partnerships with local and state-level agencies with examples drawn from four distinct aging research projects, specifically. These partnerships resulted in primary data collection with over 800 carceral constituents including prison hospice program representatives, persons who are incarcerated, and experts in correctional health and art therapies. Lessons learned and opportunities for future partnerships are also described.

